# Evaluating the accuracy of facial expressions as emotion indicators across contexts in dogs

**DOI:** 10.1007/s10071-021-01532-1

**Published:** 2021-08-02

**Authors:** A. Bremhorst, D. S. Mills, H. Würbel, S. Riemer

**Affiliations:** 1 Division of Animal Welfare, DCR-VPHI, Vetsuisse Faculty, University of Bern, 3012 Bern, Switzerland; 2 School of Life Sciences, University of Lincoln, Lincoln, LN6 7DL UK; 3 Graduate School for Cellular and Biomedical Sciences (GCB), University of Bern, 3012 Bern, Switzerland

**Keywords:** Dog, Facial expressions, Emotions, DogFACS, Diagnostic accuracy, Replication

## Abstract

Facial expressions potentially serve as indicators of animal emotions if they are consistently present across situations that (likely) elicit the same emotional state. In a previous study, we used the Dog Facial Action Coding System (DogFACS) to identify facial expressions in dogs associated with conditions presumably eliciting positive anticipation (expectation of a food reward) and frustration (prevention of access to the food). Our first aim here was to identify facial expressions of positive anticipation and frustration in dogs that are context-independent (and thus have potential as emotion indicators) and to distinguish them from expressions that are reward-specific (and thus might relate to a motivational state associated with the expected reward). Therefore, we tested a new sample of 28 dogs with a similar set-up designed to induce positive anticipation (positive condition) and frustration (negative condition) in two reward contexts: food and toys. The previous results were replicated: Ears adductor was associated with the positive condition and Ears flattener, Blink, Lips part, Jaw drop, and Nose lick with the negative condition. Four additional facial actions were also more common in the negative condition. All actions except the Upper lip raiser were independent of reward type. Our second aim was to assess basic measures of diagnostic accuracy for the potential emotion indicators. Ears flattener and Ears downward had relatively high sensitivity but low specificity, whereas the opposite was the case for the other negative correlates. Ears adductor had excellent specificity but low sensitivity. If the identified facial expressions were to be used individually as diagnostic indicators, none would allow consistent correct classifications of the associated emotion. Diagnostic accuracy measures are an essential feature for validity assessments of potential indicators of animal emotion.

**Supplementary Information:**

The online version contains supplementary material available at 10.1007/s10071-021-01532-1.

## Introduction

Emotions are relatively short-term affective responses (Mendl et al. 2010) triggered by events or stimuli of personal relevance (Gygax 2017). While much evidence indicates that at least mammalian species experience emotional states (e.g., Bennett et al. 2017; Boissy et al. 2011; Caeiro et al. 2017; De Oliveira and Keeling 2018; Dolensek et al. 2020; Hintze et al. 2016), inferring which emotion an animal may be experiencing is challenging (Gähwiler et al. 2020). Triangulating information from different sources, including context and the emotion components physiology, action tendencies, and behavioural expressions (Scherer 2005), can help to infer emotional states in animals (Mills 2017). For this purpose, valid, reliable, and robust indicators of emotions need to be developed (see, e.g., De Oliveira and Keeling 2018; Finlayson et al. 2016; Hintze et al. 2016; Kuhne et al. 2014; Rius et al. 2018).

With regard to context, different emotions are presumably elicited when a reward or punisher is anticipated, delivered, omitted, or terminated (Mendl et al. 2010; Rolls 2013). Physiological measurements such as heart rate and heart rate variability (e.g., Beerda et al. 1998; Gygax et al. 2013; Zupan et al. 2016), body temperature (e.g., Moe et al. 2012; Part et al. 2014; Riemer et al. 2016; Travain et al. 2016; but see Proctor and Carder 2016), or hormone levels (e.g., Part et al. 2014) can give some information about the arousal state. Action tendencies, such as approach or avoidance, can inform about behaviour goals (Mills 2017; Scherer 2005). Finally, if specific facial or body expressions are reliably associated with a variety of situations in which a particular emotion is likely experienced, they could have potential as indicators of the respective emotional state (Paul et al. 2005).

Facial expressions are a key to identifying human emotions (see, e.g., Darwin 1872; Ekman and Rosenberg 2005; Matsumoto et al. 2008; Scherer et al. 2013) and have also been examined in animals (e.g., cows (De Oliveira and Keeling 2018; Sandem et al. 2006), pigs (Camerlink et al. 2018), sheep (Boissy et al. 2011; Reefmann et al. 2009); bonobos (Demuru et al. 2015), mice (Defensor et al. 2012), rats (Finlayson et al. 2016), cats (Bennett et al. 2017), and dogs (Bremhorst et al. 2019; Caeiro et al. 2017); for a review on facial expressions of non-human animals, see Descovich et al. 2017). Facial expressions can be considered as reflecting emotional states if they are produced regardless of contextual features whenever a particular emotional state is experienced (see Kraut and Johnston 1979; e.g., in response to emotionally competent stimuli (Caeiro et al. 2017) such as food (Kaminski et al. 2017)). Additionally, facial expressions can have communicative functions in social interactions, as particularly emphasised by the behavioural ecology view (e.g., reviewed by Crivelli and Fridlund 2018, 2019; Hess and Thibault 2009) and may for instance provide information about the signaller’s intent (e.g., Camerlink et al. 2018), relationship with the perceiver (Matsumoto et al. 2008), or potential future behaviour (Waller et al. 2017). Studies on primates have shown that facial expressions appear to be under less voluntary control than motor behaviour (as reviewed by Descovich et al. 2017). This suggests that facial expressions of (at least some) animals have potential as honest signals of internal states (see Descovich et al. 2017).

Research on facial expressions of emotions in humans has extensively used the Facial Action Coding System (FACS) for measuring facial movements in a standardised way (e.g., Ekman and Rosenberg 2005; Ekman et al. 2002). FACS is a comprehensive, anatomically based method for the systematic coding of facial expressions that are objectively described in terms of observable movements of the facial muscles (Clark et al. 2020; Parr et al. 2007; Waller et al. 2013). Various species-specific adaptations of FACS are now available (www.animalfacs.com), including FACS for dogs, referred to as DogFACS (Waller et al. 2013).

In a previous study, we used DogFACS (Waller et al. 2013) to identify facial expressions associated with positive anticipation vs frustration in a sample of Labrador retrievers (Bremhorst et al. 2019). Positive anticipation and frustration are emotional states of different valence; while positive anticipation is considered a positive emotional state (Anderson et al. 2020; Boissy et al. 2007), frustration is considered a negative emotional state (Gygax et al. 2013; McPeake et al. 2019). However, the two states are contextually related as they may be triggered in similar situations that are associated with the expectation of a reward: While positive anticipation is expected to occur prior to the delivery of an expected reward (e.g., Anderson et al. 2020; Boissy et al. 2007), this can turn into frustration when the reward or access to it is omitted, reduced, or delayed (e.g., Amsel 1958; Anderson et al. 2020; McPeake et al. 2019).

In Bremhorst et al. (2019), we used an equivalent experimental paradigm to induce positive anticipation and frustration in dogs. In the positive condition, the conditioned expectation of access to a desired food reward was used to induce positive anticipation, whereas in the negative condition, access to a visible food reward was denied to induce frustration (Bremhorst et al. 2019). We found that the positive condition was associated with a higher incidence of the Ears adductor action (DogFACS Ear Action Descriptor (EAD) 102 (Waller et al. 2013)). In the negative condition, dogs turned their ears backwards more often (Ears flattener (EAD103)) and showed more movements in the eye region (Blink: Action Unit (AU) 145) and mouth region (Lips part: AU25, Jaw drop: AU26, and Nose lick: Action Descriptor (AD) 137; Bremhorst et al. 2019). However, this study (Bremhorst et al. 2019) did not rule out the possibility that the identified expressions could potentially be limited to the specific treatment, desired goal, or motivation associated with the type of reward used (e.g., hunger associated with the acquisition of food, but not other rewards; for a related discussion, see Caeiro et al. 2017).

A key feature of emotional responses is their contextual generalisation: When different stimuli or contexts evoke the same emotion, the same behavioural expression should be generated (Anderson and Adolphs 2014; Darwin 1872). Thus, a particular emotional state, even if elicited by different types of stimuli, should have emotion-specific behavioural denominators that share commonalities across contexts. For instance, regardless of the nature of the expected reward, positive anticipation would be expected to result in similar behavioural patterns (see Spruijt et al. 2001). Indeed, such commonalities have been demonstrated in rats anticipating different types of rewards (as reviewed by Spruijt et al. 2001) and in lambs when anticipating access to both food and toys, although also some reward-specific behaviours were found (Anderson et al. 2015). In dogs, tail wagging was associated with the expectation of access to three types of rewards related to different motivations (food, human, conspecific), but the rate of tail wagging differed between the three reward types (McGowan et al. 2014).

Reliable and robust indicators of a particular emotional state should be independent of contextual variability, including the reward type expected. In the current study, we investigated facial expressions of positive anticipation and frustration in dogs across different (reward) contexts related to different motivational states. The first aim was to identify those facial expressions that generalise across contexts, and thus have potential as respective emotion indicators, and to distinguish them from expressions that are specific to the expected reward type (and thus might be linked to the reward-related motivational state). The experimental contingencies used to induce the two target emotional states were equivalent to our previous study (Bremhorst et al. 2019); the conditioned expectation of access to a reward was used to trigger positive anticipation (positive condition) and the subsequent denial of access to a visible reward was used to induce frustration (negative condition). Extending our previous study (Bremhorst et al. 2019), we used not only food but also toys as a reward (Gerencsér et al. 2018).

If dogs’ facial expressions in the positive or negative condition are context-dependent, i.e., they differ depending on the reward type expected, they would not qualify as robust indicators of positive anticipation or frustration, respectively. Conversely, if the expressions are generalisable across contexts, this would strengthen the assumption that they allow inferences about the underlying emotional state and could thus provide a basis for developing indicators of positive anticipation or frustration in dogs. Thus, if the previously identified facial expressions (Bremhorst et al. 2019) are potentially indicative of positive anticipation or frustration, rather than the motivational state associated with the reward type the dogs were awaiting,  we expected in the current study that the Ears adductor will be more common in the positive condition and Ears flattener, Blink, Lips part, Jaw drop, and Nose lick will be more common in the negative condition, regardless of the expected reward type.

The differential occurrence of facial expressions between different emotional states is a necessary but not a sufficient criterion to qualify them as valid emotion indicators. Valid emotion indicators should correctly identify the particular emotional state if it is present. Hence, they should be sensitive for this emotion and consequently be present whenever the emotion is present. Their validity is further increased if they are specific for the emotion and are therefore absent whenever the emotion is absent. Sensitivity and specificity are common basic measures used for assessing the accuracy of diagnostic tests (e.g., Patronek and Bradley 2016). Diagnostic tests serve to determine the presence or absence of a particular condition of interest (e.g., a clinical physical or mental state) given a positive or negative test result (Greiner and Gardner 2000). Diagnostic tests never perform with perfect accuracy, and some degree of uncertainty, including false-positive and false-negative results, is commonly accepted (Baeyens et al. 2019). The accuracy (i.e., validity (Greiner and Gardner 2000)) of diagnostic tests is described by their sensitivity (the ability to correctly identify the presence of the condition of interest) and specificity (the ability to correctly identify the absence of the condition of interest (Altman and Bland 1994; Baeyens et al. 2019; Patronek and Bradley 2016); for more information on applying these metrics to canine behaviour tests, see Netto and Planta 1997; Patronek and Bradley 2016; Patronek et al. 2019; Taylor and Mills 2006; van der Borg et al. 2010). Estimates of the probability that the test results are correct can be provided by predictive values. While the positive predictive value indicates how likely a positive test result is to be a true positive, the negative predictive value indicates how likely a negative result is to be a true negative (Greiner and Gardner 2000; Parikh et al. 2008).

We can liken our potential emotion indicators to diagnostic tests and assess them using the same methods (as has been considered for animal welfare indicators (Phythian et al. 2011)). The “diagnosis” of an emotional state depends on the presence (a positive test) or absence (a negative test) of its indicator (e.g., a specific behavioural expression) in a given situation. Our second aim in the current study was to evaluate the sensitivity, specificity, and positive and negative predictive values of facial correlates that can be considered potential facial emotion indicators of positive anticipation or frustration, i.e., expressions that were associated with the positive or negative condition in the current study regardless of the type of reward expected.

## Methods

### Ethical consideration

The experiment was approved by the College of Science Research Ethics Committee, University of Lincoln (UK) (CoSREC304) and the cantonal authority for animal experimentation, the Veterinary Office of the Canton of Bern (Switzerland) (Licence number BE62/18).

### Subjects

Twenty-eight pet dogs were tested (27 Labrador retrievers and one Labrador cross with a Labrador-like morphology; 14 females and 14 males; age range: 1–14 years, mean age = 5.50 years; see Table SI 1 for further details) that were recruited personally and via social media. The owners gave their written informed consent prior to the study.

### Experimental procedure

The study was conducted in an experimental room (5.20 × 3.40 m) at the Vetsuisse campus of the University of Bern (CH). Using a within-subject design, dogs were tested in a reward anticipation and frustration test when expecting a desired reward to be delivered from an apparatus. Two reward types were used, food and toys; however, dogs show individual variation in responsiveness to food and toys (Gerencsér et al. 2018) and have preferences within both types (Pullen et al. 2010; Riemer et al. 2018; Vicars et al. 2014). Since emotional states are likely to be elicited by stimuli of personal relevance (Gygax 2017), we only used rewards for each individual that she or he was motivated to have, as determined by initial preference tests. Pilot studies showed that most dogs preferred food to toys. Therefore, to limit possible negative carry-over effects, the toy condition always preceded the food condition (in both the preference tests and the reward anticipation and frustration test). Before the first preference test, dogs could freely explore the experimental room for approximately 10 min to habituate to the situation.

### Preference tests

#### Toy preference test

From a collection of commercial dog toys differing in shape, colour, texture, size, with or without a squeaker (but no food-dispensing toys), the owner was asked to select two toys that she or he thought the dog would like. The selected toys were then given to the dog, one at a time, to see whether the dog was motivated to pick them up. If this was not the case, the toys were exchanged until two toys were found that the dog was motivated to interact with. At the beginning of each trial, the dog was held at a predefined starting point between the legs of the standing owner who closed the eyes to avoid cueing. The experimenter crouched down 1.20 m in front of the dog (Fig. SI 1) and presented both toys with extended arms for 5 s before placing them on the ground to her left and right. The positioning of the toys in her hands was balanced; each toy had to be in each hand in five trials, the order being random. The experimenter then went two steps back, closed her eyes to avoid cueing, and verbally signalled the owner to release the dog. The dog was free to make a choice (i.e., pick up a toy) and could then keep the selected toy for approximately 30 s and play with the owner (the other toy was removed immediately after the choice). After the owner returned the toy to the experimenter, a new trial started.

Ten trials were conducted. The more frequently selected toy was considered as the preferred toy of an individual. If both toys were selected equally often, an additional trial was performed and the chosen toy in this trial was used for testing. Dogs that made a choice in at least 8 of the 10 trials were considered sufficiently motivated for toys (*N* = 25) and therefore participated in the toy condition of the subsequent reward anticipation and frustration test. Three dogs were excluded from the toy preference test due to a lack of motivation to pick up the toys (Table SI 1).

#### Food preference test

The food preference test followed the same procedure as the toy preference test, but with two food rewards (cheese and sausage) presented on a white plate each. Before testing, motivation to consume both food types was assessed by giving the dog one piece of each to consume. None of the subjects were food deprived for this study. All dogs (*N* = 28) made a choice in at least 8 of 10 trials and were therefore considered sufficiently food motivated to participate in the food condition of the reward anticipation and frustration test.

#### Preferred toy vs. food preference test

With the 25 subjects that were sufficiently motivated for both reward types, an additional preference test between the individually preferred toy and food reward was performed, using the same procedure as in the previous preference tests. Since all but two dogs preferred the food to the toy reward (Table SI 1), this was not further analysed.

### Reward anticipation and frustration test

#### Experimental set-up

**Fig. 1:**
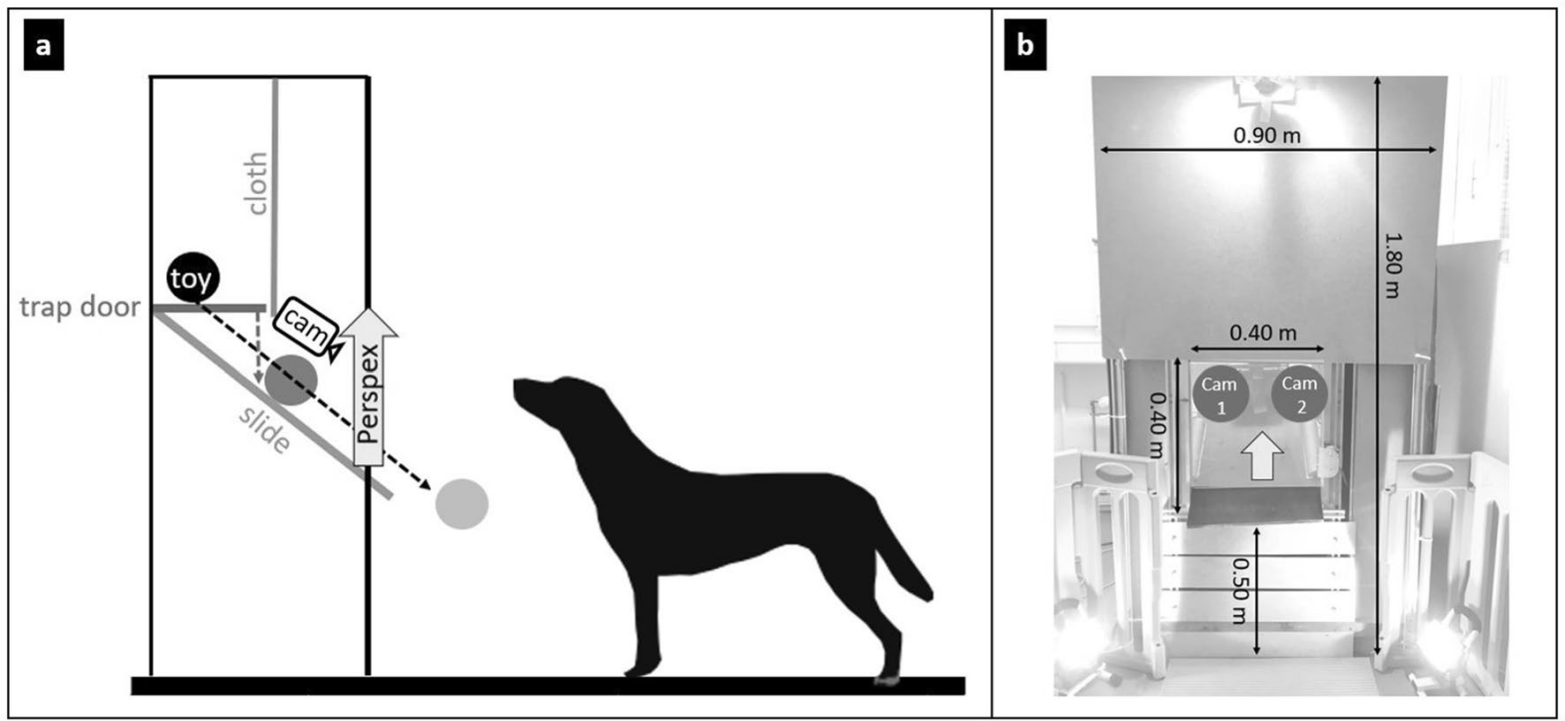
Experimental apparatus: **a** Schematic image from the side; **b** picture from a frontal view with measurements

A custom-made wooden-metal apparatus (1.80 × 0.90 m, Fig. 1) functioned as an automatic reward dispenser. When activated remotely, a trap door inside the apparatus released the reward (which until then was hidden behind a cloth to prevent the dogs from seeing it). The reward fell onto a slide that was connected to a central opening 50 cm above the floor (i.e., the approximate head height of Labrador retrievers; Fig. 1). The opening could be covered by a remotely controlled transparent Perspex panel; when the panel moved upwards, the reward fell out of the apparatus and became accessible to the dog.

At the beginning of each trial, the dog’s (and owner’s) starting point was 1.80 m from the apparatus (Fig. SI 2). The owner was sitting on a chair, wearing sunglasses to prevent inadvertent cueing and ignored the dog until the reward became accessible. Two cameras (GoPro Hero 7) in the apparatus recorded the dogs’ faces.

#### Toy condition

**Toy training**. Dogs that were sufficiently toy motivated in the toy preference test (*N* = 25) were trained to approach the apparatus and to wait there until the toy was delivered after a 5-s delay. Before the first training trial, the dog was given the toy for about 30 s to see if she or he was still interested to interact with it. A trial started after the owner sat down on the chair with the dog next to her/him. The dog observed how the experimenter hid the reward in the apparatus, which pilot trials had shown to facilitate learning. Then, the experimenter walked behind a wooden partition, located behind the dog and the owner, and remotely activated the closing of the Perspex panel. Once the opening was completely blocked, the owner released the dog using a visual (hand movement) and verbal signal. In the first five trials (in the case of a second training session in the first three), the owner then walked to the apparatus and looked through the opening to draw the dog’s attention to the apparatus. From the sixth trial on, the owner remained seated on the chair.

Together with the release signal, the experimenter initiated the automatic reward delivery; hence, 5 s later, the Perspex moved upwards and the trap door released the toy, which fell down the slide and out the apparatus through the opening. The dog could then interact with the toy and play with the owner for approximately 30 s. After the owner returned the toy to the experimenter, a new trial started using the same procedure.

To qualify for testing in the toy condition, the dogs had to meet the following training criterion: in five consecutive trials, they immediately approached the apparatus upon release and remained focused on it until the reward was dispensed. To determine when this was the case, each trial was evaluated in which the owner remained seated on the chair upon the release signal. This also allowed us to assess whether the dog was still motivated for the toy. Dogs are neophilic and seem to get easily ‘bored’ with toys (Bradshaw et al. 2015; Kaulfuß and Mills 2008), hence, toys often elicit a high response level in the first few minutes of exposure, which then quickly decreases (see Tarou and Bashaw 2007). To avoid such loss of interest or fatigue, only ten training trials per session and a maximum of two training sessions were conducted. If the training criterion had not been reached by the second session (*N* = 4) or if the response levels diminished over repeated trials (*N* = 6, including the two oldest subjects Nr. 24 and 25 in Table SI 1), the toy condition was terminated. Fifteen dogs maintained their motivation and reached the required training criterion within a mean of 8.06 evaluated trials.

**Fig. 2:**
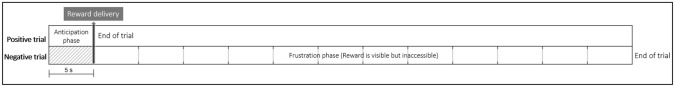
Illustration of the anticipation phase of a positive trial and the frustration phase of a negative trial

**Toy test**. Testing with the toy reward was performed in a separate session. Eleven trials were conducted, including ten positive trials and one negative trial. Positive trials followed the same procedure as the previously described training trials. In the test, the owner approached the apparatus after the release signal only in the first trial, whereas afterwards, she/he remained seated on the chair. We refer to the 5-s delay until the reward delivery as the anticipation phase (Fig. 2). The sixth trial was a negative trial. The procedure was the same as in the positive trials; however, the Perspex did not move upwards after the reward was dispensed. Thus, the dogs could see the toy inside the apparatus but not access it for 60 s (i.e., the frustration phase, Fig. 2). Five additional positive trials were performed after the negative trial to reduce possible carry-over effects of the negative experience with the apparatus.

#### Food condition

**Food training**. All dogs (*N* = 28) were sufficiently food motivated in the food preference test and were therefore trained with the food reward. The procedure was the same as described for the toy training. Before the first training trial, each dog was given one piece of the preferred food to see whether she/he was still motivated to eat it. The training criterion was reached in the first training session by 24 dogs and in the second session by two dogs (mean number of evaluated training trials to achieve the training criterion = 5.27). The two oldest subjects (subject Nr. 24 and 25 in Table SI 1) did not meet the training criterion after the second training session and were excluded from the study, because they also did not meet the training criterion in the toy condition.

**Food test**. The procedure of the food test was the same as that of the toy test, i.e., five positive trials were followed by one negative trial and another five positive trials.

### Video samples’ preparation for the subsequent DogFACS coding

Sample preparation followed the procedure of Bremhorst et al. (2019). Two positive and two negative samples of 3 s each were prepared from selected trials of the toy condition (if applicable) and the food condition, using Avidemux (version 2.6.1). The samples were prepared from the two positive trials directly preceding the negative trial by cutting out the middle 3 s of the anticipation phase. The two negative samples of each condition were taken from the frustration phase of the negative trial. We randomly selected the starting point of each negative sample (using the R random number generator, function ‘sample’, repetitions excluded). However, the first 10 s were excluded as the frustration response may not immediately set in. Our collection of negative samples therefore comprised different time points of the frustration phase, to account for possible fluctuations in the dogs’ expression during the longer negative trial. In each sample, the dog’s face had to be visible for at least 2 s. If this was not the case, the next preceding trial (for the positive samples) or another starting second (for the negative samples) was selected. A total of 164 video samples were generated [toy condition: 30 positive and 30 negative samples (*N* = 15; N refers to the number of dogs); food condition: 52 positive and 52 negative samples (*N* = 26)).

### DogFACS coding

A certified DogFACS coder coded the video samples according to the DogFACS manual (Waller et al. 2013; www.animalfacs.com). All upper face action units (Inner brow raiser (AU101), Eye closure (AU143), Blink (AU145)), all lower face action units (Nose wrinkler and Upper lip raiser (AU109 + 110), Upper lip raiser (AU110), Lip corner puller (AU12), Lower lip depressor (AU116), Lip pucker (AU118), Lips part (AU25), Jaw drop (AU26), Mouth stretch (AU27)), all action descriptors (Tongue show (AD19), Blow (AD34), Suck (AD35), Lip wipe (AD37), Nose lick (AD137)), and four ear action descriptors (Ears forward (EAD101), Ears adductor (EAD102), Ears flattener (EAD103), Ears downward (EAD105); Ears rotator (EAD104) was excluded, because according to the DogFACS manual (Waller et al. 2013), this ear movement cannot be produced by dogs with floppy ears such as Labrador retrievers) were coded as present or absent in the positive and negative samples. The coder was unaware of the study aims, hypotheses, and procedure. To determine the neutral ear position, which was required for the EAD coding, the same images as in our previous study (Bremhorst et al. 2019) were used. Coding was performed using the Solomon Coder software (version 15.03.15, Andràs Péter).

Reliability coding of thirty randomly selected samples (> 15% of all samples) was performed by a second certified DogFACS coder for the 12 final DogFACS variables that were present in at least 10% of (at least) either the positive or the negative condition (i.e., Inner brow raiser, Blink, Upper lip raiser, Lip corner puller, Lower lip depressor, Lips part, Jaw drop, Tongue show, Nose lick, Ears adductor, Ears flattener, Ears downward; see Table SI 2). DogFACS variables with a lower prevalence (i.e., Eye closure, Nose wrinkler and Upper lip raiser, Lip pucker, Mouth stretch, Blow, Suck, Lip wipe, Ears forward; see Table SI 2) were not included in the analyses as their value as a potential emotion indicator would be low. Cohen’s Kappa was calculated in RStudio 1.0.153 (package psych (Revelle 2019)) and demonstrated at least substantial (i.e., Cohen’s Kappa ≥ 0.61 (Landis and Koch 1977)) intercoder agreement for all variables (Cohen’s Kappa range: 0.63–1.00; Table SI 2).

### Statistical analyses

#### Facial correlates of positive anticipation and frustration

Statistical analyses were performed in RStudio (version 1.0.153). Binomial mixed-effect models (GLMER, R-package “lme4” (Bates et al. 2014)), with Type III sum of squares, were used to assess the effect of the fixed factors (1) condition (positive/negative), (2) reward type (food/toy) and (3) the interaction between condition and reward type on the 12 final DogFACS variables (each was used as an individual response variable). Subject ID was included as a random factor to account for multiple observations of the same individual and thus dependency in the data set. Subject sex and age were used as covariates. For the model computation, data from 15 dogs in the toy condition (30 positive and 30 negative samples) and from 26 dogs in the food condition (52 positive and 52 negative samples) were used. Graphical visualisations (Figs. 4, 6 and 7) were done with Tableau Software (Version 2019.1). Facial expressions with a significant effect of condition but no effects of reward type or the reward type*condition interaction are subsequently referred to as positive correlates (when significantly more common in the positive condition) or negative correlates (when significantly more common in the negative condition).

When balancing the risk for type I and II statistical errors, we prioritised reducing the risk of falsely rejecting a potentially promising response (type-II-error, false negative) over the risk of falsely accepting a variable (type-I-error, false positive). Whereas the former could cause a variable to be excluded from any further examination for the development of indicators of positive anticipation or frustration in dogs, in the latter case, we expect that the falsely accepted variables will be identified as lacking predictive validity in subsequent studies. Thus, we did not correct for multiple testing (as recommended by Bender and Lange (2001) for exploratory studies).

#### Diagnostic accuracy assessment

**Fig. 3:**
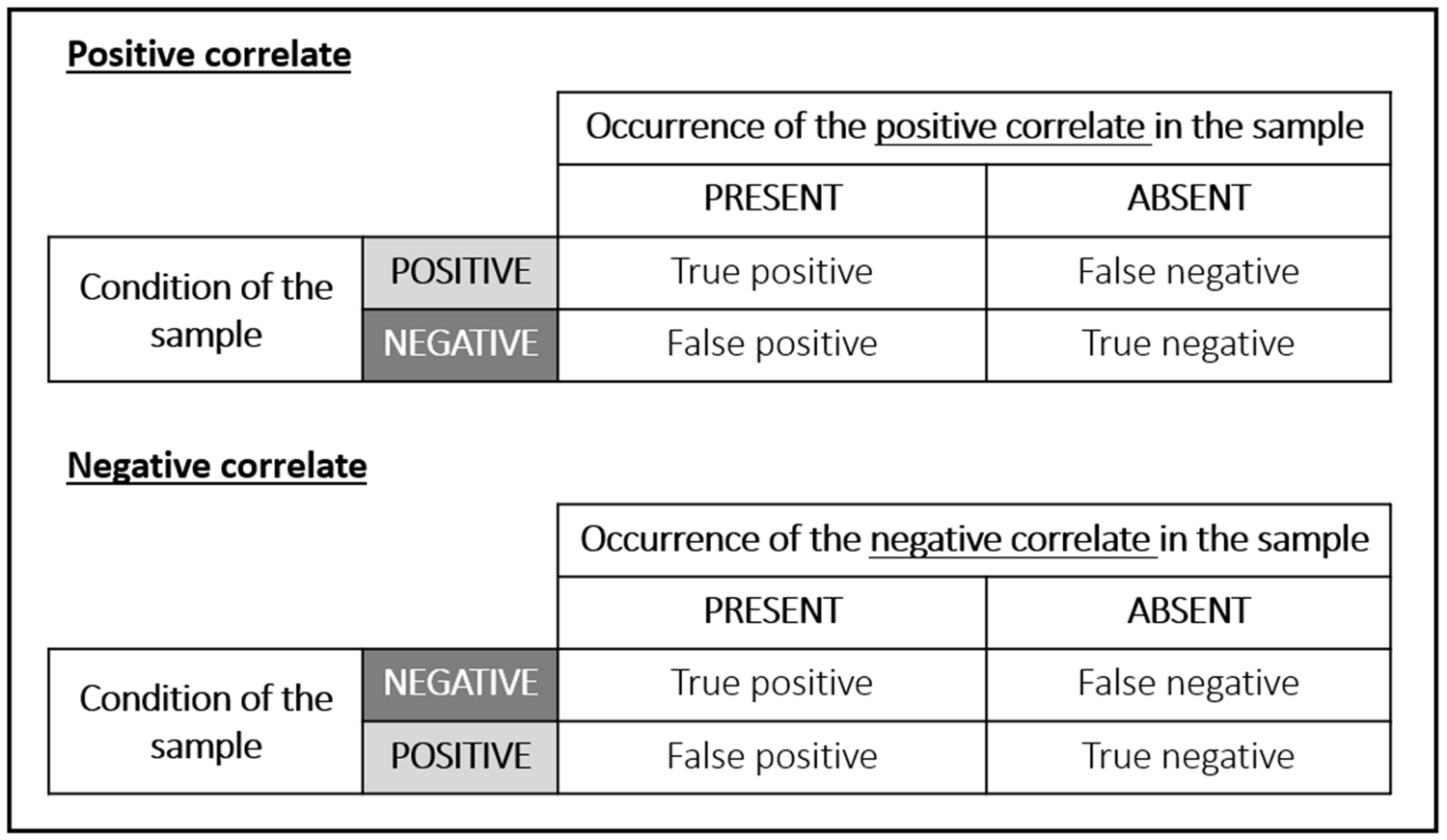
2 × 2 contingency tables showing the four outcomes used for classifying the frequencies of presence/absence of the positive and negative correlates in the positive and negative samples

Diagnostic accuracy was assessed for the positive and negative correlates, since they could have the potential to serve as emotion indicators. We first calculated the frequencies of the presence and absence of these positive and negative correlates in the positive and negative samples and classified them as true positive, false positive, true negative, or false negative (Fig. 3).

The frequencies of true positives, false positives, true negatives, and false negatives of the positive and negative correlates were then used to calculate the sensitivity, specificity, and positive and negative predictive values using the following standard formula:

$$\mathrm{Sensitivity}=\frac{\mathrm{True positives}}{(\mathrm{True positives}+\mathrm{False negatives})}$$

$$\mathrm{Specificity}=\frac{\mathrm{True negatives}}{(\mathrm{True negatives}+\mathrm{False positives})}$$

$$\mathrm{Positive predictive value}=\frac{\mathrm{True positives}}{(\mathrm{True positives}+\mathrm{False positives})}$$

$$\mathrm{Negative predictive value}=\frac{\mathrm{True negatives}}{(\mathrm{True negatives}+\mathrm{False negatives})}.$$

For interpreting the calculated estimates, the following guidelines were used (as per Briggs-Gowan et al. (2004) and Cicchetti et al. (1995) for sensitivity and specificity): below 0.70 = poor, 0.70–0.79 = fair; 0.80–0.89 = good, and 0.90–1.00 = excellent.

## Results

### Facial correlates of positive anticipation and frustration

**Fig. 4:**
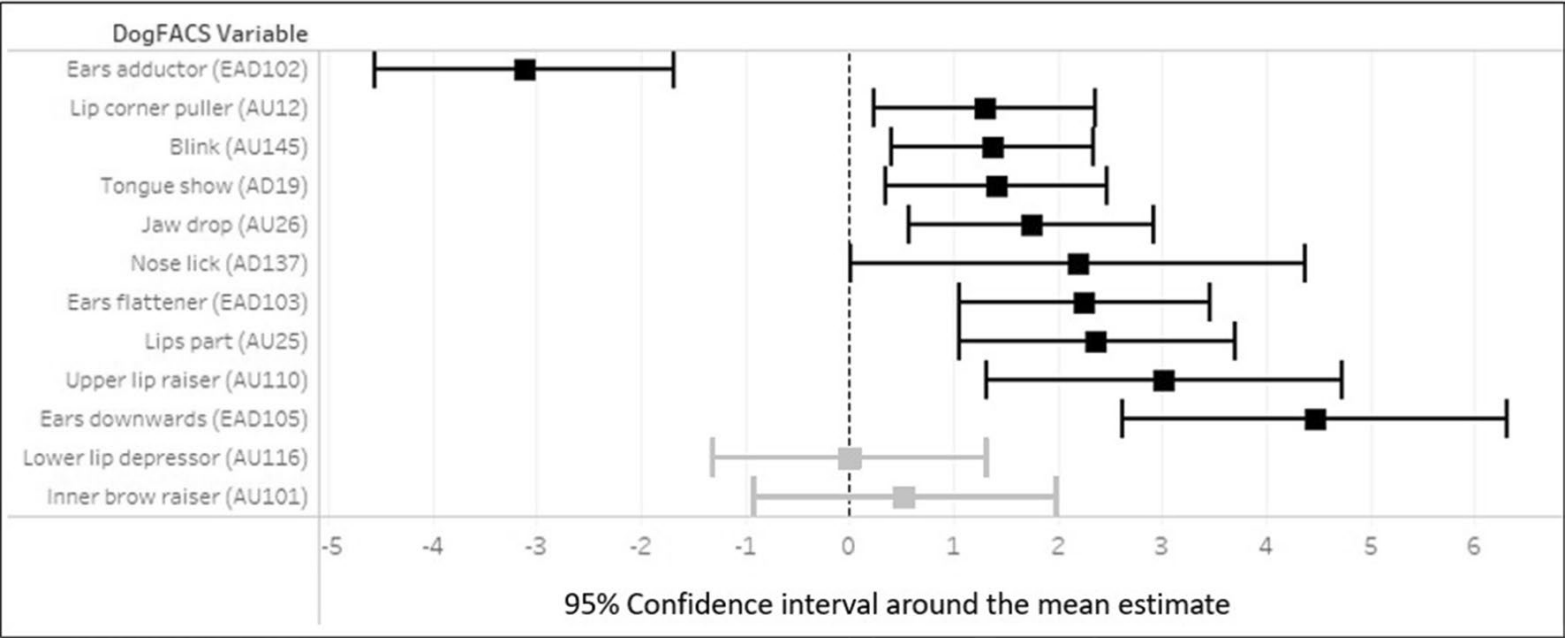
The 95% CI around the mean estimates for the twelve final DogFACS variables with a significant (black) or non-significant (grey) effect of condition

Binomial mixed-effect models demonstrated a significant effect of condition on 10 of the 12 final DogFACS variables (Table SI 3). The only variable that was more common in the positive compared to the negative condition was Ears adductor (*χ*^2^_1_ = 18.20, *p* ≤ 0.001; Fig. 4, Table SI 3). Nine variables occurred more frequently in the negative condition, namely Blink (*χ*^2^_1_ = 7.74, *p* = 0.005), Ears flattener (*χ*^2^_1_ = 13.52, *p* ≤ 0.001), Ears downward (*χ*^2^_1_ = 22.63, *p* ≤ 0.001), Lips part (*χ*^2^_1_ = 12.46, *p* ≤ 0.001), Jaw drop (χ^2^_1_ = 8.58, *p* = 0.003), Tongue show (*χ*^2^_1_ = 6.77, *p* = 0.009), Nose lick (*χ*^2^_1_ = 3.90, *p* = 0.05), Lip corner puller (*χ*^2^_1_ = 5.83, *p* = 0.02), and Upper lip raiser (*χ*^2^_1_ = 12.05, *p* ≤ 0.001; Fig. 4, Table SI 3).

**Fig. 5:**
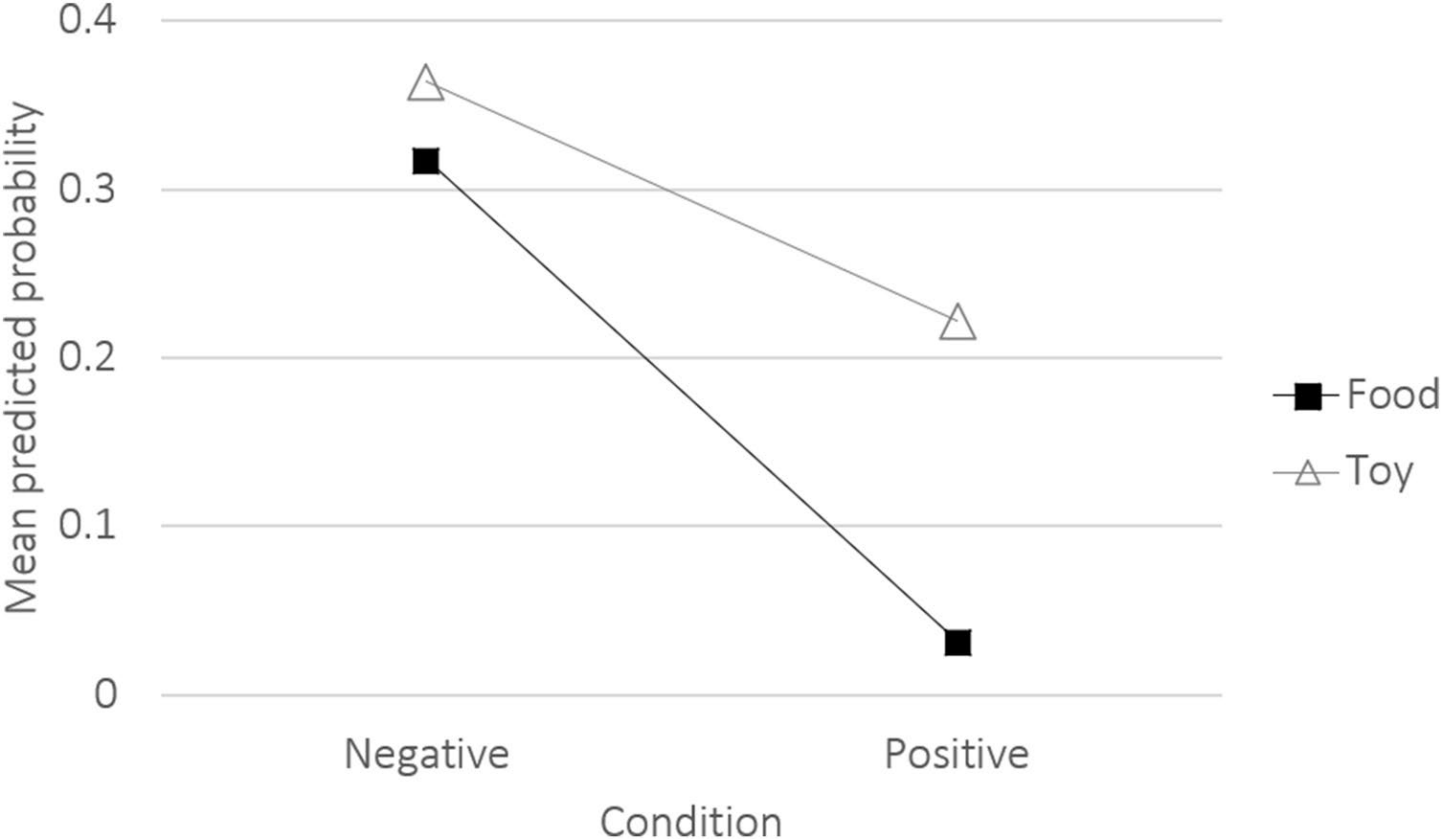
Mean predicted probabilities of the Upper lip raiser for the interaction effect of condition (positive/negative) and reward type (food/toy)

The Upper lip raiser was the only variable that was significantly affected by reward type (*χ*^2^_1_ = 5.41, *p* = 0.02; Table SI 3) and where a significant interaction between reward type and condition was found (*χ*^2^_1_ = 4.22, *p* = 0.04; Fig. 5; Table SI 3). Hence, since Ears adductor did not differ significantly depending on the reward type expected, it was considered as a positive correlate, and Blink, Ears flattener, Ears downward, Lips part, Jaw drop, Tongue show, Nose lick, and Lip corner puller were considered as negative correlates.

### Diagnostic accuracy assessment

**Fig. 6:**
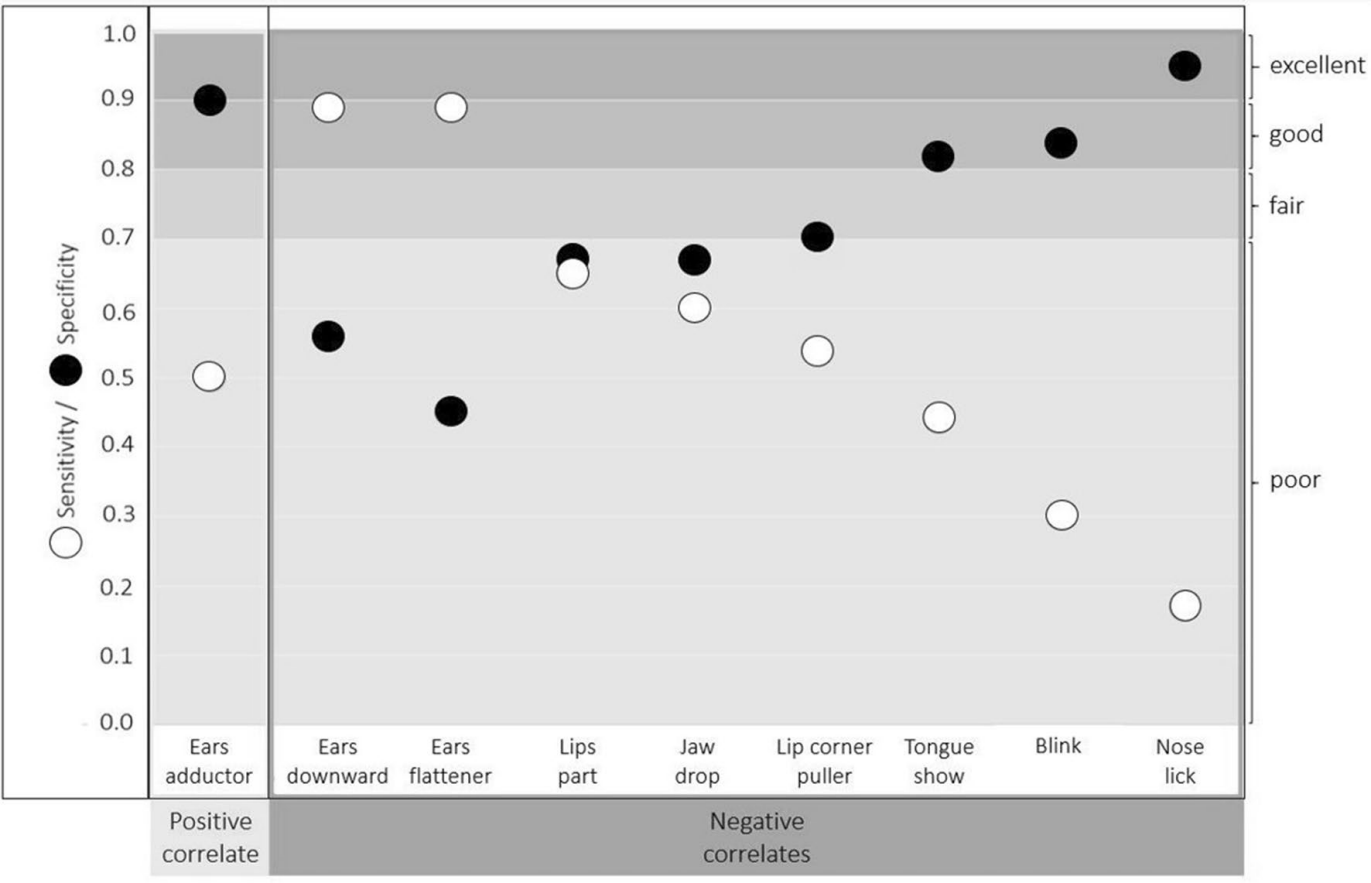
Sensitivity (white circles) and specificity (black circles) of the positive correlate (Ears adductor) and the negative correlates (Ears downward, Ears flattener, Lips part, Jaw drop, Lip corner puller, Tongue show, Blink, and Nose lick; the negative correlates are sorted in descending order of sensitivity)

**Fig. 7:**
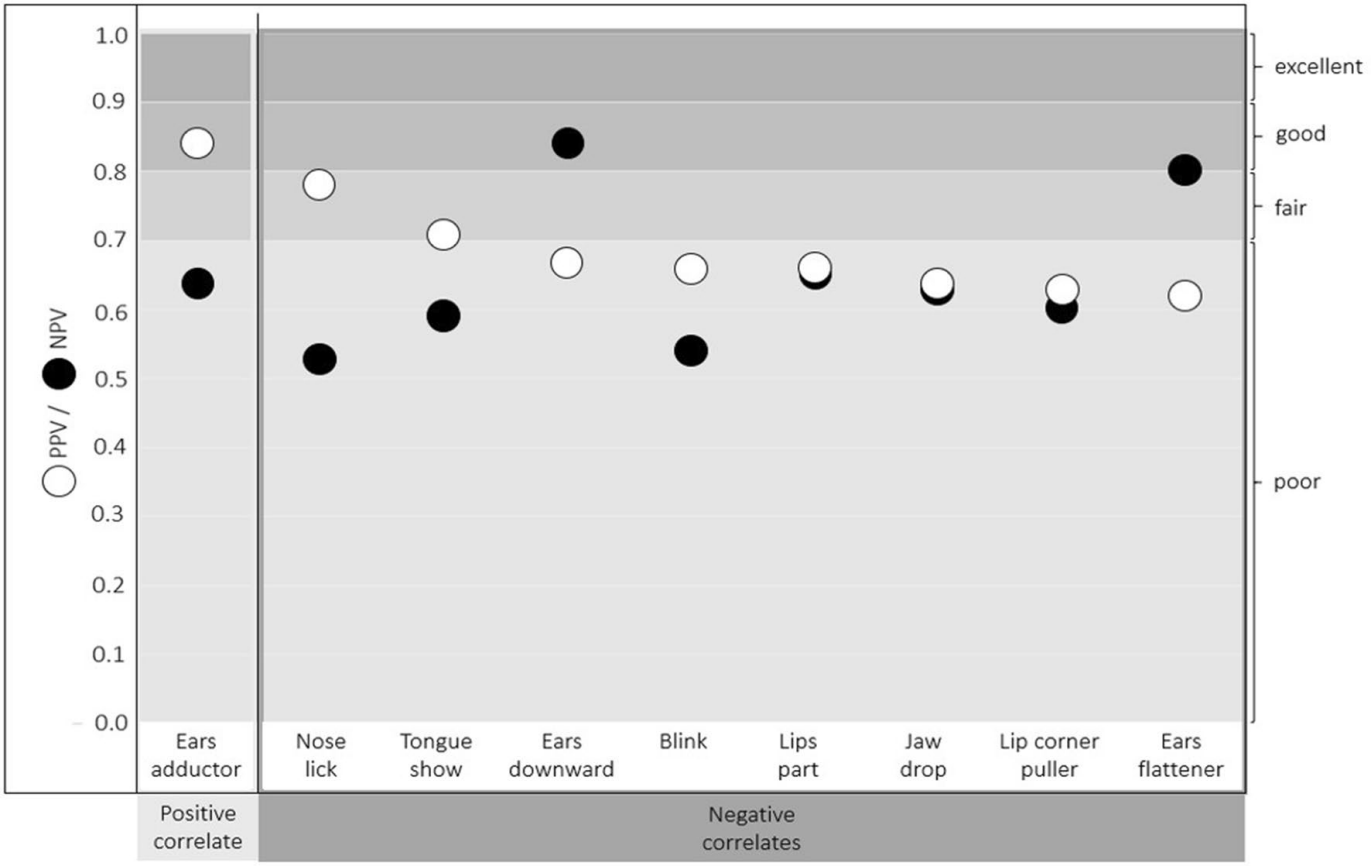
Positive predictive value (“PPV”; white circles) and negative predictive value (“NPV”; black circles) of the positive correlate (Ears adductor) and the negative correlates (Nose lick, Tongue show, Ears downward, Blink, Lips part, Jaw drop, Lip corner puller, and Ears flattener; the negative correlates are sorted in descending order of the positive predictive value)

The calculated frequencies of true-positive, true-negative, false-positive, and false-negative results were used to calculate the sensitivity, specificity, and positive and negative predictive value of the positive and negative correlates (Table SI 4). The positive correlate Ears adductor had poor sensitivity (0.50) but excellent specificity for the positive condition (0.90; Fig. 6, Table SI 4), a good positive predictive value (0.84) but a poor negative predictive value (0.64; Fig. 7, Table SI 4).

Six of the eight negative correlates had poor sensitivity for the negative condition (range: 0.17–0.65), the exceptions were the two negative ear actions (Fig. 6, Table SI 4). Ears flattener and Ears downward had good sensitivity (both 0.89; Fig. 6, Table SI 4). However, the specificity of Ears flattener and Ears downward was poor and the lowest of all negative correlates (0.45 and 0.56; Fig. 6, Table SI 4). The positive predictive value of Ears flattener and Ears downward was also poor (0.62 and 0.67), but the negative predictive value was good (0.80 and 0.84; Fig. 7, Table SI 4). Nose lick had the lowest sensitivity of all negative correlates for the negative condition (0.17), but the highest specificity (0.95; Fig. 6, Table SI 4). The positive predictive value of Nose lick was fair (0.78), but the negative predictive value was poor (0.53; Fig. 7, Table SI 4). The specificity was good for Tongue show (0.82) and Blink (0.84), fair for Lip corner puller (0.70), but poor for Lips part (0.67), and Jaw drop (0.67; Fig. 6, Table SI 4). The positive predictive value was fair for Tongue show (0.71) and poor for Blink (0.66), Lip corner puller (0.64), Lips part (0.66), and Jaw drop (0.64; Fig. 7, Table SI 4). The negative predictive values of these five negative correlates were poor (Blink: 0.55, Lip corner puller: 0.60, Lips part: 0.65, Jaw drop: 0.63, Tongue show: 0.59; Fig. 7, Table SI 4).

## Discussion

The main findings of the current study (see Table 1 for a summary) showed that dogs generally produced distinct facial expressions in situations that are likely to induce positive anticipation or frustration, respectively. As in our previous study (Bremhorst et al. 2019), the Ears adductor was more frequent in the positive condition and Blink, Ears flattener, Lips part, Jaw drop, and Nose lick were more frequent in the negative condition. Thereby, we extended the external validity of our previous findings (Bremhorst et al. 2019) with a new sample of dogs, a different test environment and apparatus, and the use of two types of rewards to elicit the target emotional states. Furthermore, four additional facial expressions (Ears downward, Tongue show, Lip corner puller, and Upper lip raiser) were more common in the negative condition of the current study. In our previous study (Bremhorst et al. 2019), Ears downward was not analysed as its prevalence was low, Tongue show and Lip corner puller did not differ significantly between the positive and negative condition, and the Upper lip raiser had an insufficient intercoder agreement.

**Table 1:**
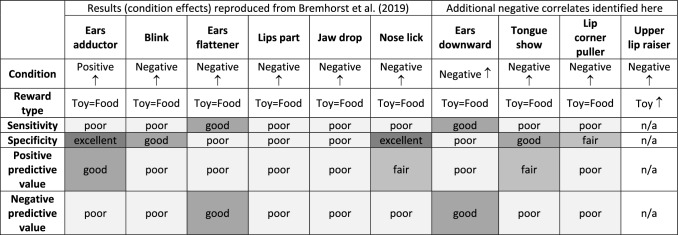
Summary of the main results of the current exploratory study (condition = significant effect of condition; reward type = significant effect of reward type; sensitivity; specificity; positive predictive value; negative predictive value)

The Upper lip raiser was the only expression that was affected by motivational context in the current study, being shown more often when a toy was expected than when the reward was food. In addition, an interaction between condition and reward type was found, indicating that in the negative condition, the dogs raised their upper lip more often when expecting the toy reward than when expecting food. The Upper lip raiser, therefore, appears to be context-specific, possibly reflecting toy-related motivation. This makes the Upper lip raiser unsuitable as a reliable and robust indicator of frustration. All other identified expressions (Ears adductor, Ears flattener, Ears downward, Blink, Lips part, Jaw drop, Tongue show, Nose lick, and Lip corner puller) were produced in the respective condition regardless of the expected reward type. Even though all but two dogs preferred food to the toy reward, this preference had no significant effect on dogs’ facial display. The contextual invariability makes these facial expressions potential candidate indicators of positive anticipation or frustration, respectively, in dogs.

As with other species with mobile ears (e.g., sheep (Reefmann et al. 2009); mice (Langford et al. 2010); cats (Bennett et al. 2017); cattle (De Oliveira and Keeling 2018)), the ears seem to be particularly important in conveying emotional state in dogs, since three ear movements differed between the positive and the negative condition. Ears adductor, the only positive emotion correlate, was also associated with positive anticipation in our previous study (Bremhorst et al. 2019) and in another study where dogs of different breeds and mixes were examined in more variable everyday settings (Caeiro et al. 2017). Nevertheless, this upwards ear movement has rarely been studied to date and it is therefore unclear whether it is exclusively associated with positive anticipation. Since the Ears adductor was not more common during the putative state of happiness in an earlier study (Caeiro et al. 2017), it does not seem to be a generic correlate of positively valenced states in dogs. Erect ears may, however, also be associated with attention (Darwin 1872). An increase in attention is a main characteristic of anticipation (Spruijt et al. 2001). However, also dogs that appeared to be vigilant in potentially fearful situations have been described to hold their ears up, but turned backwards at the base (Gähwiler et al. 2020). Further studies are needed to assess the production of the Ears adductor in dogs in a wider range of positive, but also negative emotional settings to better understand its function in dogs’ expressive display.

The antagonistic ventral movement of the ear pinnae, Ears downward, as well as Ears flattener were more common in the negative condition. The Ears flattener (i.e., backwards-directed ears) has been suggested to be associated with appeasement, submission, fear, anxiety, and stress in dogs (although empirical evidence supporting these functions is not always provided) (e.g., Beerda et al. 1998; Firnkes et al. 2017; Flint et al. 2018; Gähwiler et al. 2020; Landsberg et al. 2015; Schilder and Van Der Borg 2004; Siniscalchi et al. 2018; Tami and Gallagher 2009). Therefore, the available evidence suggests that flattened ears are frequently associated with negatively valenced states, and thus, this expression could be suitable for developing indicators of negative emotions in dogs. It is unclear whether this also applies to Ears downward, since this ear action has not received much attention to date; so further research is needed that examines this ear movement in a range of different (emotional) contexts to systematically determine its function.

In line with our previous study (Bremhorst et al. 2019), blinking was increased in the negative condition relative to the positive condition. Blinking has previously been associated with fear states in dogs (Gähwiler et al. 2020; Mills 2005). Additionally, blinking has been considered to be an appeasement gesture, which dogs produce in conflicting situations (but empirical evidence validating this function is lacking) (e.g., Kuhne 2016; Kuhne et al. 2012; Siniscalchi et al. 2018). In other species, blinking has also been associated with emotional states (e.g., in cats (Humphrey et al. 2020) and humans (Harris et al. 1966; Porter and Ten Brinke 2008)), but also with impulsivity (e.g., in horses (as reviewed by McBride et al. 2017)), stress and arousal (in humans (Wood and Saunders 1962)). The occurrence of increased blinking in dogs during different contexts associated with putatively negative states suggests that it could be a facial correlate of negatively valenced emotions, but alternatively it could also be a generic stress/arousal correlate.

All identified mouth actions were more common in the negative condition. Jaw drop and Lips part are usually shown in combination, and both accompany most other mouth actions, including the additionally identified Tongue show and Nose lick. They also form part of other composite mouth actions including panting, yawning, barking, and biting. Nose lick has been observed in situations linked to different potentially negative emotional states in dogs (e.g., Albuquerque et al. 2018; Bremhorst et al. 2019; Firnkes et al. 2017; Flint et al. 2018; Kuhne 2016; Stellato et al. 2017; but see Gähwiler et al. 2020), and it has also been associated with stress or arousal (e.g., Beerda et al. 1997; Rehn and Keeling 2011). Tongue show, which can be a component of panting, has also been suggested to be linked to stress in dogs (Kaminski et al. 2017). Likewise, the Lip corner puller was suggested to communicate stress in dogs (as reviewed by Siniscalchi et al. 2018; note that they used the term “long lips” which appears to conform to the DogFACS Lip corner puller).

Since most of the facial correlates identified in the current study have previously also been reported to occur in situations that are likely to trigger emotional states other than positive anticipation or frustration in dogs, these behaviours may not be exclusive to the emotional states studied here, but may be more general valence or arousal correlates. An exception may be the antagonistic ear movements Ears adductor and Ears downward, which have so far only been empirically associated with positive anticipation or frustration, respectively, in dogs. However, both ear movements have received little attention in canid research so far, and so there is a lack of data to associate them exclusively with these two emotional states. An increase in arousal during the longer negative condition could potentially lead to increased use of some facial expressions and hence might explain why more actions were associated with the negative condition than with the positive condition. While no measures of the physiological arousal level were collected in the current study, unpublished analyses of the dogs’ body language in the current study suggest the opposite; arousal seems to decrease over the course of the negative trial, as indicated by a reduction in tail wagging rate and lowering of tail height as the trial progressed. Future studies are needed that examine positive anticipation and frustration in dogs in a wider range of contexts but also other emotional states. Furthermore, behaviour measures should be triangulated with physiological parameters of arousal to allow systematically distinguishing correlates of putative emotional states from more generic stress/arousal correlates.

None of the positive or negative correlates would have enabled consistent correct designations of the associated positive or negative condition if they had been used as emotion indicators on their own. Only the Ears flattener and Ears downward had high sensitivity for their corresponding (negative) condition, while the other correlates were more specific than sensitive. Ears flattener and Ears downward were present in approximately 89% of the negative samples, so their sensitivity was good (few false negatives). However, since they also occurred in 55% (Ears flattener) or 44% (Ears downward) of the positive samples (false positives), a relatively high rate of positive samples would have been incorrectly classified as negative (poor specificity). Such an inverse relationship of sensitivity and specificity is common for diagnostic tests: as sensitivity increases, specificity decreases and vice versa (Parikh et al. 2008; Patronek and Bradley 2016). The positive predictive values of Ears flattener and Ears downward were poor, with more than a third of the positive results being false positives. Conversely, the negative predictive values of the two negative ear actions were good, with 80% (Ears flattener) and 84% (Ears downward) of the negative results being true negatives.

Ears adductor had excellent specificity, occurring almost exclusively in positives samples. However, since it occurred in only half of the positive samples, its sensitivity was poor. The positive predictive value of the Ears adductor was good, with 84% of positive results being true positives. However, since around 36% of the negative results were false negatives, the negative predictive value of the Ears adductor was poor. Highly specific tests are rarely positive in the absence of the condition they indicate (Kyriacou 2001). Consequently, the presence of the Ears adductor could have some indicative value for identifying the positive condition, as is typical for highly specific tests (see Baeyens et al. 2019). Nonetheless, half of the cases from the positive condition could remain undetected, without recourse to further measures.

Tongue show and Nose lick also had good or excellent specificity (few false positives), but sensitivity and negative predictive values were poor. The proportion of true positives among all positive results were 71% (Tongue show) and 78% (Nose lick), respectively, and so their positive predictive values were fair. Thus, when Tongue show and Nose lick are observed, this could have some indicative value for inferring the negative condition. Nonetheless, since both actions were only present in 17% (Nose lick) and 44% (Tongue show) of the negative samples, many negative samples would remain undetected if they were used as individual indicators of frustration. Lip corner puller and Blink also had fair or good specificity, respectively, and poor sensitivity. While this suggests that their presence may be indicative of the negative condition, both have poor positive and negative predictive values. Lips part and Jaw drop had poor sensitivity, specificity, and predictive values. Taken together, even though these variables differed significantly between the negative and positive condition, they do not seem to be very promising candidates for the development of frustration indicators in dogs.

The high prevalence of the negative correlate Ears flattener in the positive condition was rather unexpected, given that this ear movement was previously associated with negative emotional states in dogs (e.g., Gähwiler et al. 2020). Although we lack reference values for its specificity for putatively negative emotional states, this result potentially challenges our assumption that we consistently induced the target emotional states as intended in the respective conditions. Positive anticipation and frustration are closely linked, and positive anticipation can shift to frustration (Anderson et al. 2020). We cannot exclude that a transition from the positive to the negative emotional state may have occurred already during the anticipation phase, even though it was kept short (5 s). Frustration tolerance can vary between individual dogs (McPeake et al. 2019; Turcsán et al. 2018), and the positive condition could have been appraised differently by different subjects (see Mendl et al. 2010). Consequently, frustration may have set in faster in some individuals than in others.

Frustration may furthermore occur when individuals are lacking control over a situation (Elder and Menzel 2001). In a previous study with dogs, access to a reward was either dependent on the completion of a trained operant task or independent of the subjects’ actions (McGowan et al. 2014). Whereas dogs in the first condition showed behaviours interpreted as indicating positive emotional states, dogs in the second condition who could not actively control access to the reward showed behaviours interpreted as indicating frustration (McGowan et al. 2014). Our subjects might have perceived a lack of control not only in the negative but also in the positive condition, since they could not actively influence the delivery of the reward. Future studies could increase the level of controllability and predictability for the dogs in the positive condition, e.g., by enabling them to control access to the reward by performing an operant behaviour. Testing dogs with such a modified positive condition would provide insights not only for evaluating the validity of our treatments, but also for assessing whether giving the dogs more control changes their facial expressions and how this affects the respective accuracy estimates.

To reduce the possible impact of morphological variation on dogs’ facial expressions, only Labrador retrievers were tested in the current study; thus, breed-specific differences in expression cannot be ruled out. However, a previous study explored effects of cephalic type, ear morphology, jowl length, and breed on dogs’ facial expressions and found that only two DogFACS variables, Upper lip raiser (AU110) and Lip corner puller (AU12), were affected by jowl length (Caeiro et al. 2017). Furthermore, dogs with erect ears, but not dogs with floppy ears, can rotate their ears laterally and externally (DogFACS Ears rotator (Waller et al. 2013)). None of these three actions appeared to be promising emotion indicators in the current study (although the Lip corner puller was identified as a negative correlate here, its diagnostic accuracy was relatively low). So far, no effect of morphology has been reported for those expressions identified here as promising potential indicators of positive anticipation or frustration in dogs. Nonetheless, the assessment of the external validity and generalisability of the present results requires future studies with a greater variety of dogs.

Diagnostic accuracy assessments have received little attention in research on animal emotions. While they can complement analyses of associations between emotional states and behavioural expressions, as exemplified here, by providing an objective approach to evaluating the validity of potential emotion indicators, they also have limitations. For instance, sensitivity and specificity of a given indicator commonly vary between studies, since they can be influenced by a range of factors, including differences between populations and sampling methods, but also systematic and random errors (Greiner and Gardner 2000). Furthermore, sensitivity, specificity, and predictive values can be affected by the prevalence of the condition of interest in the sample (e.g., Baeyens et al. 2019; Greiner and Gardner 2000; Patronek and Bradley 2016; Taylor and Mills 2006). Typically, assessments of diagnostic accuracy for a test under evaluation are compared to a gold standard, which is a reference test with high accuracy for the condition of interest (Greiner and Gardner 2000; Parikh et al. 2008; Patronek and Bradley 2016). However, we lack gold standards for indicators of emotional states that can be applied to dogs, and so different alternative approaches for estimating the accuracy of diagnostic tests have been suggested, including statistical methods (as reviewed by Enøe et al. 2000) or the determination of an expert consensus (Phythian et al. 2011; Rutjes et al. 2007). The latter has been exemplified for the development of pain indicators in cats (Merola and Mills 2016), and it could be a useful approach in the development of emotion indicators in dogs, as well. Nonetheless, the use of diagnostic accuracy measures in the current study constitutes a useful approach to evaluate the validity of behaviour correlates of affect more widely in animal emotion and welfare research.

## Supplementary Information

Below is the link to the electronic supplementary material.

Supplementary file1 (XLSX 21 KB)

Supplementary file2 (DOCX 464 KB)

## Data Availability

The dataset generated during the current study is provided as supplementary material.
