# Supplementary material for: Evaluating the accuracy of facial expressions as emotion indicators across contexts in dogs
Source: Anim Cogn. 2021 Aug 2;25(1):121–36. doi: 10.1007/s10071-021-01532-1 (PMC8904359; doi:10.1007/s10071-021-01532-1)
Supplement: Supplementary file 2 — Supplementary file2 (DOCX 464 KB) [file 10071_2021_1532_MOESM2_ESM.docx]

# Supplementary Information (SI)

# Evaluating the accuracy of facial expressions as emotion indicators across contexts in dogs

Animal Cognition, Springer

# Bremhorst, A.^1,2,3,*^, Mills, D.S.^2^, Würbel, H.^1^, Riemer, S.^1^

^1^ Division of Animal Welfare, DCR-VPHI, Vetsuisse Faculty, University of Bern, 3012 Bern, Switzerland

^2^ School of Life Sciences, University of Lincoln, Lincoln, LN6 7DL, United Kingdom

^3^ Graduate School for Cellular and Biomedical Sciences (GCB), University of Bern, 3012 Bern, Switzerland

*Corresponding author: annika.huber@vetsuisse.unibe.ch

**Table 1.** Details on the subjects and experimental parts. Preference tests: columns indicate for each dog whether the motivation for the respective reward type was sufficient or insufficient. The ‘Preferred reward’ column shows the most frequently chosen reward when given the choice between the individually preferred food vs. preferred toy. Reward anticipation and frustration test: columns indicate whether the training criterion to proceed to testing with the respective reward type was reached.

| **Subject** | **Sex** | **Age**  **(years)** | **Preference tests** | | | **Reward anticipation and frustration test** | |
| --- | --- | --- | --- | --- | --- | --- | --- |
|  |  |  | **Toy motivation** | **Food motivation** | **Preferred reward** | **Training criterion – Toy** | **Training criterion  – Food** |
| 1 | M | 1 | Sufficient | Sufficient | Food | Reached | Reached |
| 2 | F | 6.5 |  |  |  |  |  |
| 3 | F | 2 |  |  |  |  |  |
| 4 | M | 6.5 |  |  |  |  |  |
| 5 | M | 4 |  |  |  |  |  |
| 6 | F | 4 |  |  |  |  |  |
| 7 | M | 7 |  |  |  |  |  |
| 8 | F | 3.5 |  |  |  |  |  |
| 9 | M | 5.5 |  |  |  |  |  |
| 10 | M | 4.5 |  |  |  |  |  |
| 11 | M | 2 |  |  |  |  |  |
| 12 | M | 5.5 |  |  |  |  |  |
| 13 | M | 3 |  |  |  |  |  |
| 14 | F | 8 |  |  | Toy |  |  |
| 15 | M | 3.5 |  |  |  |  |  |
| 16 | F | 6.5 |  |  | Food | Not reached |  |
| 17 | M | 2.5 |  |  |  |  |  |
| 18 | F | 4.5 |  |  |  |  |  |
| 19 | F | 1.5 |  |  |  |  |  |
| 20 | F | 3 |  |  |  |  |  |
| 21 | M | 3.5 |  |  |  |  |  |
| 22 | F | 3 |  |  |  |  |  |
| 23 | F | 12.5 |  |  |  |  |  |
| 24 | F | 14 |  |  |  |  | Not reached |
| 25 | F | 13.5 |  |  |  |  |  |
| 26 | F | 9.5 | Insufficient * |  | NA | NA ⱡ | Reached |
| 27 | M | 6.5 |  |  |  |  |  |
| 28 | M | 7 |  |  |  |  |  |

*No motivation to pick up the toys.

ⱡ Not trained and tested with this reward type due to insufficient motivation in the preference test.

**Table 2.** Prevalence of all coded DogFACS variables in the positive and negative samples and Cohen’s Kappa determined for DogFACS variables with a prevalence ≥10% in either condition.

| **DogFACS variables** | **Prevalence** | | **Cohen’s Kappa** |
| --- | --- | --- | --- |
|  | **Positive samples** | **Negative**  **samples** |  |

| **Final DogFACS variables (sufficient prevalence)** |
| --- |

| Inner brow raiser (AU101) | 0.90 | 0.93 | 0.84 |
| --- | --- | --- | --- |
| Blink (AU145) | 0.16 | 0.30 | 0.63 |
| Upper lip raiser (AU110) | 0.11 | 0.34 | 0.87 |
| Lip corner puller (AU12) | 0.30 | 0.54 | 0.76 |
| Lower lip depressor (AU116) | 0.16 | 0.22 | 0.78 |
| Lips part (AU25) | 0.33 | 0.65 | 0.93 |
| Jaw drop (AU26) | 0.33 | 0.60 | 0.86 |
| Tongue show (AD19) | 0.18 | 0.44 | 0.83 |
| Nose lick (AD137) | 0.05 | 0.17 | 0.84 |
| Ears adductor (EAD102) | 0.50 | 0.10 | 0.81 |
| Ears flattener (EAD103) | 0.55 | 0.89 | 1.00 |
| Ears downward (EAD105) | 0.44 | 0.89 | 0.66 |

| **Excluded DogFACS variables (insufficient prevalence)** |
| --- |

| Eye closure (AU143) | 0.00 | 0.00 | NA |
| --- | --- | --- | --- |
| Nose wrinkler and Upper lip raiser (AU109+110) | 0.00 | 0.02 |  |
| Lip pucker (AU118) | 0.01 | 0.04 |  |
| Mouth stretch (AU27) | 0.01 | 0.09 |  |
| Blow (AD34) | 0.01 | 0.02 |  |
| Suck (AD35) | 0.01 | 0.04 |  |
| Lip wipe (AD37) | 0.00 | 0.04 |  |
| Ears forward (EAD101) | 0.00 | 0.02 |  |

**Table 3.** Results of the binomial logistic regression models showing the effects of condition (level: negative), reward type (level: toy) and the interaction between condition and reward type (level: negative x toy) on the occurrence of the twelve final DogFACS variables. Bolded effects were significant (P ≤ 0.05).

| **Response factor** | **Predictor** | **R^2^** | **df** | **χ^2^** | **Estimate** | **SE** | **z** | **CI** | | **P** |
| --- | --- | --- | --- | --- | --- | --- | --- | --- | --- | --- |
|  |  |  |  |  |  |  |  | **2.5%** | **97.5%** |  |
| Variable more common in the positive condition | | | | | | | | | | |
| Ears adductor | **Condition** | 0.47 | **1** | **18.20** | **-3.12** | **0.73** | **-4.27** | **-4.55** | **-1.68** | **<0.001** |
|  | Reward |  | 1 | 0.00 | 0.01 | 0.55 | 0.02 | -1.07 | 1.09 | 0.98 |
|  | Condition x Reward |  | 1 | 0.68 | 0.80 | 0.97 | 0.83 | -1.10 | 2.71 | 0.41 |
| Variables more common in the negative condition | | | | | | | | | | |
| Blink | **Condition** | 0.14 | **1** | **7.74** | **1.38** | **0.49** | **2.78** | **0.41** | **2.35** | **0.005** |
|  | Reward |  | 1 | 0.05 | 0.14 | 0.64 | 0.21 | -1.11 | 1.39 | 0.83 |
|  | Condition x Reward |  | 1 | 3.44 | -1.65 | 0.89 | -1.86 | -3.39 | 0.09 | 0.06 |
| Ears flattener | **Condition** | 0.51 | **1** | **13.52** | **2.25** | **0.61** | **3.68** | **1.05** | **3.46** | **<0.001** |
|  | Reward |  | 1 | 0.35 | 0.34 | 0.58 | 0.59 | -0.80 | 1.48 | 0.56 |
|  | Condition x Reward |  | 1 | 1.08 | 1.32 | 1.27 | 1.04 | -1.17 | 3.81 | 0.30 |
| Ears downward | **Condition** | 0.66 | **1** | **22.63** | **4.47** | **0.94** | **4.76** | **2.63** | **6.31** | **<0.001** |
|  | Reward |  | 1 | 0.78 | 0.61 | 0.69 | 0.89 | -0.74 | 1.96 | 0.38 |
|  | Condition x Reward |  | 1 | 0.47 | -0.79 | 1.15 | -0.69 | -3.05 | 1.47 | 0.49 |
| Lips part | **Condition** | 0.64 | **1** | **12.46** | **2.38** | **0.67** | **3.53** | **1.06** | **3.70** | **<0.001** |
|  | Reward |  | 1 | 0.28 | 0.38 | 0.71 | 0.53 | -1.01 | 1.76 | 0.59 |
|  | Condition x Reward |  | 1 | 0.35 | 0.58 | 0.98 | 0.59 | -1.34 | 2.50 | 0.55 |
| Jaw drop | **Condition** | 0.59 | **1** | **8.58** | **1.75** | **0.60** | **2.93** | **0.58** | **2.92** | **0.003** |
|  | Reward |  | 1 | 0.35 | 0.40 | 0.67 | 0.59 | -0.92 | 1.71 | 0.56 |
|  | Condition x Reward |  | 1 | 0.54 | 0.67 | 0.92 | 0.73 | -1.13 | 2.48 | 0.46 |
| Tongue show | **Condition** | 0.40 | **1** | **6.77** | **1.41** | **0.54** | **2.60** | **0.35** | **2.48** | **0.009** |
|  | Reward |  | 1 | 0.004 | 0.04 | 0.67 | 0.06 | -1.28 | 1.36 | 0.95 |
|  | Condition x Reward |  | 1 | 0.46 | 0.57 | 0.85 | 0.68 | -1.09 | 2.24 | 0.50 |
| Nose lick | **Condition** | 0.20 | **1** | **3.90** | **2.20** | **1.11** | **1.98** | **0.02** | **4.38** | **0.05** |
|  | Reward |  | 1 | 1.77 | 1.60 | 1.21 | 1.33 | -0.76 | 3.96 | 0.18 |
|  | Condition x Reward |  | 1 | 0.62 | -1.07 | 1.36 | -0.79 | -3.74 | 1.59 | 0.43 |
| Lip corner puller | **Condition** | 0.49 | **1** | **5.83** | **1.30** | **0.54** | **2.41** | **0.24** | **2.36** | **0.02** |
|  | Reward |  | 1 | 0.00 | -0.01 | 0.64 | -0.02 | -1.26 | 1.24 | 0.99 |
|  | Condition x Reward |  | 1 | 0.51 | 0.61 | 0.85 | 0.71 | -1.06 | 2.28 | 0.48 |
| Upper lip raiser | **Condition** | 0.41 | **1** | **12.05** | **3.02** | **0.87** | **3.47** | **1.32** | **4.73** | **<0.001** |
|  | **Reward** |  | **1** | **5.41** | **2.12** | **0.91** | **2.33** | **0.33** | **3.91** | **0.02** |
|  | **Condition x Reward** |  | **1** | **4.22** | **-2.20** | **1.07** | **-2.05** | **-4.29** | **-0.10** | **0.04** |
| Variables that did not differ between conditions | | | | | | | | | | |
| Inner brow raiser | Condition | 0.24 | 1 | 0.52 | 0.53 | 0.74 | 0.72 | -0.92 | 1.99 | 0.47 |
|  | Reward |  | 1 | 0.03 | 0.15 | 0.95 | 0.16 | -1.72 | 2.03 | 0.87 |
|  | Condition x Reward |  | 1 | 0.17 | -0.53 | 1.31 | -0.41 | -3.10 | 2.03 | 0.68 |
| Lower lip depressor | Condition | 0.40 | 1 | 0.00 | 0.00 | 0.67 | 0.00 | -1.31 | 1.31 | 1.00 |
|  | Reward |  | 1 | 1.69 | 0.93 | 0.71 | 1.30 | -0.47 | 2.32 | 0.19 |
|  | Condition x Reward |  | 1 | 1.28 | 1.08 | 0.95 | 1.13 | -0.79 | 2.94 | 0.26 |

**Table 4.** Frequencies of the presence and absence of the positive and negative correlates in the positive and negative samples with the respective classifications and the calculated sensitivity, specificity, positive predictive value, and negative predictive value.

| **DogFACS variable** | **Samples** | **Present/**  **absent** | **Classification** | **Freq.** | **Sensitivity** | **Specificity** | **Positive predictive value** | **Negative predictive value** |
| --- | --- | --- | --- | --- | --- | --- | --- | --- |
| Positive correlate | | | | | | | | |
| Ears adductor | Positive | Present | True positive | 41 | 0.50 | 0.90 | 0.84 | 0.64 |
|  | Negative | Present | False positive | 8 |  |  |  |  |
|  | Positive | Absent | False negative | 41 |  |  |  |  |
|  | Negative | Absent | True negative | 74 |  |  |  |  |
| Negative correlates | | | | | | | | |
| Blink | Negative | Present | True positive | 25 | 0.30 | 0.84 | 0.66 | 0.55 |
|  | Positive | Present | False positive | 13 |  |  |  |  |
|  | Negative | Absent | False negative | 57 |  |  |  |  |
|  | Positive | Absent | True negative | 69 |  |  |  |  |
| Ears flattener | Negative | Present | True positive | 73 | 0.89 | 0.45 | 0.62 | 0.80 |
|  | Positive | Present | False positive | 45 |  |  |  |  |
|  | Negative | Absent | False negative | 9 |  |  |  |  |
|  | Positive | Absent | True negative | 37 |  |  |  |  |
| Ears downward | Negative | Present | True positive | 73 | 0.89 | 0.56 | 0.67 | 0.84 |
|  | Positive | Present | False positive | 36 |  |  |  |  |
|  | Negative | Absent | False negative | 9 |  |  |  |  |
|  | Positive | Absent | True negative | 46 |  |  |  |  |
| Lips part | Negative | Present | True positive | 53 | 0.65 | 0.67 | 0.66 | 0.65 |
|  | Positive | Present | False positive | 27 |  |  |  |  |
|  | Negative | Absent | False negative | 29 |  |  |  |  |
|  | Positive | Absent | True negative | 55 |  |  |  |  |
| Jaw drop | Negative | Present | True positive | 49 | 0.60 | 0.67 | 0.64 | 0.63 |
|  | Positive | Present | False positive | 27 |  |  |  |  |
|  | Negative | Absent | False negative | 33 |  |  |  |  |
|  | Positive | Absent | True negative | 55 |  |  |  |  |
| Tongue show | Negative | Present | True positive | 36 | 0.44 | 0.82 | 0.71 | 0.59 |
|  | Positive | Present | False positive | 15 |  |  |  |  |
|  | Negative | Absent | False negative | 46 |  |  |  |  |
|  | Positive | Absent | True negative | 67 |  |  |  |  |
| Nose lick | Negative | Present | True positive | 14 | 0.17 | 0.95 | 0.78 | 0.53 |
|  | Positive | Present | False positive | 4 |  |  |  |  |
|  | Negative | Absent | False negative | 68 |  |  |  |  |
|  | Positive | Absent | True negative | 78 |  |  |  |  |
| Lip corner puller | Negative | Present | True positive | 44 | 0.54 | 0.70 | 0.64 | 0.60 |
|  | Positive | Present | False positive | 25 |  |  |  |  |
|  | Negative | Absent | False negative | 38 |  |  |  |  |
|  | Positive | Absent | True negative | 57 |  |  |  |  |


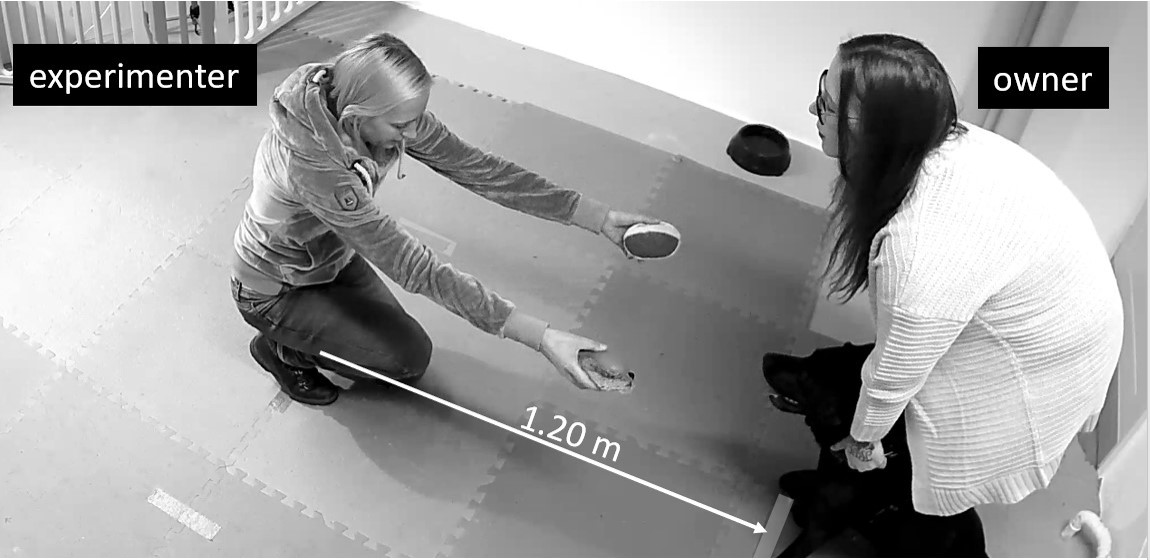


**Fig. 1** Presentation of the two toys in the toy preference test (schematic measures and marks were added for illustration in this image).


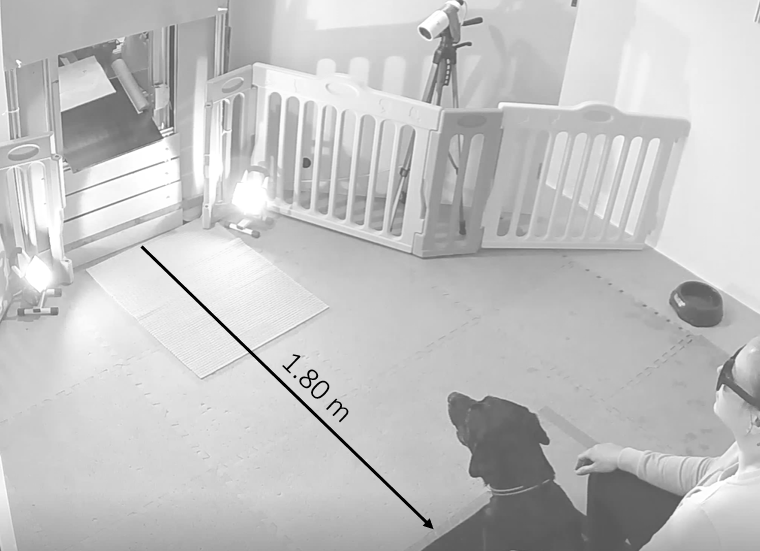


**Fig. 2** Dog and owner in the starting position of the reward anticipation and frustration test. The experimenter was located behind them behind a wooden partition.
